# The Impact of Histological Annotations for Accurate Tissue Classification Using Mass Spectrometry Imaging

**DOI:** 10.3390/metabo11110752

**Published:** 2021-10-30

**Authors:** Juliana Pereira Lopes Gonçalves, Christine Bollwein, Anna Melissa Schlitter, Benedikt Martin, Bruno Märkl, Kirsten Utpatel, Wilko Weichert, Kristina Schwamborn

**Affiliations:** 1 Institute of Pathology, School of Medicine, Technical University of Munich, Trogerstraße 18, 81675 Munich, Germany; juliana.goncalves@tum.de (J.P.L.G.); christine.bollwein@tum.de (C.B.); melissa.schlitter@tum.de (A.M.S.); wilko.weichert@tum.de (W.W.); 2 German Cancer Consortium (DKTK), Partner Site Munich, 80336 Munich, Germany; 3 General Pathology and Molecular Diagnostics, Medical Faculty, University Hospital of Augsburg, 86156 Augsburg, Germany; benediktm@googlemail.com (B.M.); bruno.maerkl@uka-science.de (B.M.); 4 Institute of Pathology, University of Regensburg, 93053 Regensburg, Germany; Kirsten.Utpatel@klinik.uni-regensburg.de; 5 Comprehensive Cancer Center Munich (CCCM), Marchioninistraße 15, 81377 Munich, Germany

**Keywords:** mass spectrometry imaging, proteomics, histological annotations, supervised classification, on-tissue analysis

## Abstract

Knowing the precise location of analytes in the tissue has the potential to provide information about the organs’ function and predict its behavior. It is especially powerful when used in diagnosis and prognosis prediction of pathologies, such as cancer. Spatial proteomics, in particular mass spectrometry imaging, together with machine learning approaches, has been proven to be a very helpful tool in answering some histopathology conundrums. To gain accurate information about the tissue, there is a need to build robust classification models. We have investigated the impact of histological annotation on the classification accuracy of different tumor tissues. Intrinsic tissue heterogeneity directly impacts the efficacy of the annotations, having a more pronounced effect on more heterogeneous tissues, as pancreatic ductal adenocarcinoma, where the impact is over 20% in accuracy. On the other hand, in more homogeneous samples, such as kidney tumors, histological annotations have a slenderer impact on the classification accuracy.

## 1. Introduction

The field of spatial proteomics has the potential to provide further understanding to fields such as biology, pharmacology, and medicine. Mass spectrometry imaging (MSI) is a technology that in the last couple of decades has been enabling mapping of molecules directly in tissue sections [1]. One of the major advantages of MSI is its compatibility with histologic annotations [1]. Because of that, and its ease of integration with the current diagnostic sample preparation processes, MSI has been moving in the direction of clinical applications [2,3]. Since its development, this technology has been addressing some unanswered questions from a clinical point of view. However, aiming to translate years of pathology investigation and experience into a more automated approach—based on mass spectrometry data collection, and data interpretation using machine learning algorithms—is a daring feat.

The different tissues in the human body have distinct histology. Despite working together, they present defined cellular and molecular compositions. The unique array of proteins, the specificity of the cell type and its location, accurately define the tissue and the organ behavior. Regarding tumor environment, its composition can also be very heterogeneous, depending on the tumor location and development, there are innumerous variables to be taken into consideration when evaluating the tissue. On top of that, in the regions neighboring tumors, other structures or other tissues can often be found, that can challenge the tissue classification. Namely, different inflammatory cells, necrotic tissue, connective tissue, blood vessels, and adipose tissue are commonly found in the vicinity of the tumors and have very different molecular profiles than the tumor [4]. That is why tumor diagnosis is a scrupulous process, which entails the identification of tumor entity and subentity, recognition of the tumor origin, analysis of predictive biomarkers, and druggable targets. This process is a responsibility usually undertaken by pathologists, where the tissue is thoroughly analyzed, through a series of staining procedures, followed by exhaustive microscopic analysis [5]. Cell type, cell density, cellular morphology, tissue origin, tissue integrity, functional state, reactive changes, and neoplastic alterations are some of the information pathologists use for the assessment of the tissue. While conveying all that information to a machine learning approach has been proven for particular diagnostic applications (e.g., detection of prostate cancer in needle core biopsies by Paige Prostate, an FDA-approved artificial intelligence-based software solution (www.paige.ai, accessed on 28 October 2021)) [6], it might still be some years away for most applications, or might not even be feasible.

To build reliable predictors for all given tumors and reactive conditions, the input information has to be premeditated. For instance, to distinguish tumor tissue from healthy tissue, we need to teach the model instructing what is tumor and what is healthy tissue by providing accurate information on those tissue regions. However, at this point that is the only information the model has. Thus, it cannot determine the tumor origin or prognosis because it simply does not possess that information to make the decision. On the other hand, if we teach the machine to distinguish between tumor types, we cannot expect the outcome to tell us about the normal tissue. Different models need to be built according to the information we would like to obtain. This applies to all fields of machine learning including image analysis. For instance, Campanella et al. used the same convolutional neural network (Resnet34) for the task of detecting prostate cancer infiltrates in needle core biopsies of the prostate, basal cell carcinoma in skin resection specimens and breast cancer infiltrates in axillary lymph nodes based on histopathological slide images [6]. Nevertheless, the neural network had to be trained separately for the specific cancer type to generate unique models for the detection of prostate cancer, basal cell carcinoma and breast cancer, respectively. However, even the restriction to the tumor entity might be too imprecise considering tumor subtypes, grading, and also the tumor environment (primary vs. metastasis). This illustrates that an algorithm must be tailored specifically to the task at hand.

Similar to image analysis, MSI, measuring hundreds of spectra per single tumor core, originates large datasets, with information that amounts to the whole proteome, which possibly cannot be fully interpreted. For that reason, the more specific and well thought our hypothesis is, the more likely we are to find answers. To that extent, histopathological annotation, meaning the process of evaluation of detailed images of stained tissue sections by a trained pathologist and marking the regions of interest, is crucial. This reflects the assessment of morphological and architectural features that characterize the tissue and its pathological changes and entails a complex mixture of visual motifs and cellular properties that allow the pathologist to infer about the disease. The accuracy of this process is essential for diagnostic as well as research purposes. That is why histological annotations can help narrow down the obtained answers to more meaningful results. The importance of histological annotations has been previously acknowledged, but its relevance for tissue classification using machine learning algorithms has never been quantified [7,8,9].

To quantitatively compare the classification accuracy with and without histological annotations, we have measured the peptide/protein content of trypsin digested samples of five different patient cohorts using matrix-assisted laser desorption/ ionization time of flight (MALDI-TOF) MSI. The measured cohorts were then used to train three of the most commonly employed classification algorithms (LDA—linear discriminant analysis, RF—random forest, and SVM—support vector machine) before and after histological annotations. These classification algorithms are all based on supervised learning, hence the input information, which is required for building the model, is subsequently used in the decision making in the classification process.

As tumor heterogeneity differs depending on the tissue origin, we have investigated different entities. Renal tumor samples (including clear cell renal cell carcinoma—ccRCC, papillary renal cell carcinoma—pRCC, chromophobe renal cell carcinoma—chRCC, renal oncocytoma—RO, and angiomyolipoma—AML), which present a more uniform tissue composition, colon cancer (CC) which presents a medium tissue complexity, pancreatic ductal adenocarcinoma (PDAC), and cholangiocarcinoma (CCC), which are usually more heterogeneously composed. The models were then employed for the classification of a subset of the data, and accuracy values were compared.

## 2. Results

**Figure 1:**
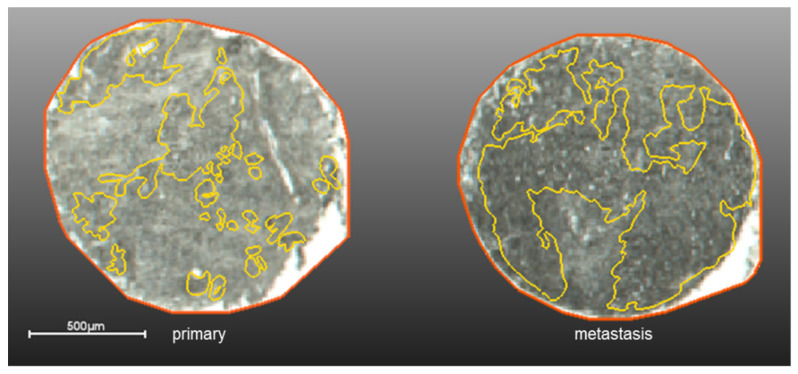
Example of histological annotation (in yellow) on the PDAC primary (**left**) and metastasis (**right**) TMA overlaid with the measurement regions (orange).

To evaluate the impact of histological annotations we have considered 6 different tissue microarrays (TMAs), with a total of 354 samples from patients of four different pathology institutes. Based on the hematoxylin and eosin (H&E) staining of the measured samples, the epithelial regions were carefully annotated by a pathologist. For the annotations either SCiLS Cloud (which is now a discontinued service previously provided by Bruker) or QuPath were used. The annotated regions were then co-registered with the measurement regions using SCiLS Lab (Figure 1).

The datasets were divided in training and validation (70% of the measured regions) and test set (30%). The average accuracies (Table 1, Table 2, Table 3, Table 4 and Table 5) of each classification model were obtained from the confusion matrix of the test set. To allow for a fair comparison, all models were fitted using the same classification algorithms and without tuning.

Duplicates of samples from 60 patients (total of 120 tissue cores) diagnosed with renal cell carcinoma were combined in a TMA with tumor tissue and normal tissue neighboring the tumor regions. Renal cell carcinoma (RCC) was selected as it presents a more homogeneous setting with only little stroma. The classification accuracy was evaluated on the models’ capability to differentiate normal from tumor tissue (Table 1).

Pancreatic ductal adenocarcinoma (PDAC) displays a more heterogeneous tissue composition with various amounts of intermingled stroma and inflammatory cells, and often presents distant metastasis at the time of diagnosis [10]. Therefore, we have evaluated the impact of histological annotation of the tumor regions in pairs of primary and metastasis collected from 17 patients (Table 2).

CCC, a tumor that exhibits an aggressive growth and low rate of recovery, being therefore one of the most lethal cancer types [11,12], was also evaluated by itself, by comparing tumor tissue with healthy tissue (Table 3). Here we have considered 145 tissue cores from 51 patients.

Current employed approaches for Union for International Cancer Control (UICC) staging I/II colon cancer (CC) are insufficient for reliable prognosis prediction [13]. Here the different staging attributed on diagnosis was compared before and after histological annotations (Table 4). By choosing to compare such small proteomic changes, we are also challenging the approach and evaluating the impact of histological annotations for convoluted changes in the tissue. This TMA is composed by 78 tissue cores from 41 patients.

PDAC and CCC have comparable tissue complexity and high histomorphological resemblance [14]. We have therefore evaluated two mixed TMAs with a total of 120 tissue cores from 54 patients diagnosed with PDAC or CCC (Table 5). The use of two TMAs also introduces some experimental variability, which impacts the classification accuracy.

**Figure 2:**
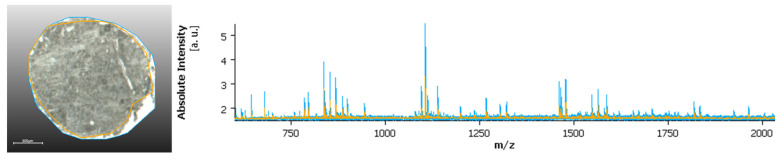
Spectral comparison: Mass spectrum of the whole measurement region (blue) and after the removal of the border regions (orange).

On a parallel note, the removal of the regions on the border of the core (for TMAs) or measurement regions, may also impact the analysis (Figure 2). Areas without tissue tend to ionize better than areas with tissue (so called edge effect), which can cause the ionization in the tissue to get lost among the higher intensity peaks from the non-tissue areas.

## 3. Discussion

As MSI is a rapidly evolving technology, it becomes essential to develop fast and reliable methods to further its applications. Using machine learning approaches to analyze the vast data from on-tissue measurements does not only provide quicker answers but also allows for deeper scrutiny of the data. However, the analysis outcome is unequivocally connected to the input data, which should be carefully curated in order to achieve meaningful results. Here we have weighed the effect of histological annotations of the measured tissue in the classification accuracy of different datasets. For that, we have utilized the very same tissue section, which after matrix removal was stained with H&E and scanned for evaluation and annotation by a pathologist.

As tissues have different cellular compositions depending on their origin and function, also tumor tissues can differ in their composition and complexity. For that reason, we have evaluated specimens with different degrees of heterogeneity (tissue heterogeneity is illustrated in more detail in the Supplementary Materials, in Figures S1–S5). From what we can see from the classification results (Table 1, Table 2, Table 3, Table 4 and Table 5), classification accuracies do not show a consistent improvement across all tumor types. Perhaps not surprisingly, tissue heterogeneity also dictates different needs when it comes to tissue annotation. More homogeneous tumor samples, such as kidney tumors (Figures S1 and S2), only benefit slightly from meticulous histological annotations, with an overall improvement of 0.4% for LDA and RF and approximately 1% for SVM. More heterogeneous tumor samples (such as the PDAC), have a more pronounced benefit from precise annotation of the histological features, with an improvement of classification accuracy of over 20%. Additionally, CCC, with a heterogeneous tissue distribution, shows an impactful improvement on the classification accuracy after detailed histological annotation.

Often datasets have more than one TMA, or different measurements need to be compared, which also introduces inter-measurement variabilities that impact the classification. Considering that, we have also tested the impact of histological annotations on a dataset composed of two mixed TMAs. By attempting to differentiate PDAC and CCC (Figure S4), we achieved an improvement comparable to the CCC (tumor vs. normal), proving once again the relevance of this step in the data preparation. We have also posed seemingly impossible questions, to challenge the hypothesis and provide deeper insight into the impact of the histological annotations. We compared CC samples with different patient outcomes, to assist in prognosis prediction, which is currently lacking for UICC stage I/II CC patients. However, only 22% of the patients had been diagnosed with stage II colon cancer, making the cohort classes unbalanced. Despite the inequality, also here we see an improvement due to histological annotations for two of the three tested models, indicating that even in most challenging cases, the use of annotated structures still benefits the accuracy of the model. Overall, we can objectively state that annotation of the histological features benefited the datasets included in this study, and therefore should be considered an integral step of data curation with the purpose of building more accurate classification models.

It is also important to stress that all samples were collected from donor blocks, where only tumor enriched regions had been pre-selected for building the TMAs. However, this does not always translate in cores only harboring tumor for two main reasons. One of the reasons can be explained by the way TMAs are constructed. In the first step, areas of interest (e.g., tumor and adjacent normal) are annotated on a stained H&E section from each donor block. These annotations are transferred to the matching area on the donor tissue block, and subsequently, cores are punched out of the donor block and placed into the preformed hole within the recipient block. Cores might not harbor the intended tissue type due to errors during annotation transfer from section to block or TMA construction itself. Another reason lies in the fact that tissue blocks are three-dimensional, and a section only captures one plane in a three-dimensional space. Thus, a TMA section might initially harbor the tissue type of interest, but as sections are cut from the block this might no longer be the case for deeper sections. The second reason is based on the tissue of interest itself. If the tissue of interest is heterogeneous, it will be heterogeneous no matter the size of the investigated area.

Another relevant note is that areas without tissue or with very low tissue content, which are often included in the measurement regions, in particular at the edge between tissue and the glass slide, can also impact the classification. These areas usually produce higher ion intensity than tissue regions, as shown in Figure 1, which can result in suppression of lower signals and further challenge the data analysis process. Histological annotation also addresses this issue, removing non-tissue areas from the data analysis, considering only tissue regions, and avoiding border regions, and where the tissue might have poorly adhered to the glass slides (thus, for example, resulting in changes in tissue height). Additionally, sample preparation can alter the shape and size of the samples, and some difficulties might arise when co-registering the measurement regions and the H&E scan. This has higher impact in more heterogeneous tissue, where more detailed annotations are required, and also in larger TMAs. To solve this predicament, software that can accommodate those changes, and provide a good overlay of the measurement regions and the annotated H&Es is required to maximize the accuracy of the analysis process. A perfect overlay is, as we see it, essential for the evaluation of more heterogeneous tissue structures. Additionally, the measurement resolution is of high relevance to the topic especially in relation to more heterogeneous samples. Routine MSI, usually carried at 30–50 µm pixel size, might not provide enough detail to accommodate smaller tissue structures. Especially when considering smaller samples, such as biopsies, where tumor content can be significantly less and therefore, a higher measurement resolution must be considered.

As spatial resolution increases towards single cell level, it will become very time consuming, especially for routine measurements, to carry out manual annotation. In such cases, employing digital pathology, that can facilitate and expedite the annotation of samples, could be the solution for further advancement of the technology.

MSI is setting itself to solve the most challenging conundrums in pathology, there are, however, a few things that the scientific and medical community need to establish before the technology leaps to standard practice. Gathering vast sample pools that include adequate diversity per tumor type/ subtype, collect detailed patient data, treatment and outcome information, and thoroughly annotated specific tumor regions are some of the steps required to achieve highly accurate classification models.

## 4. Materials and Methods

### 4.1. Sample Selection and TMA Preparation

The cohorts were identified by searching the administrative database of the respective pathology institutes (PDAC and CCC from the Institute of Pathology, University of Regensburg; CC from the Institute of Pathology, University of Augsburg; PDAC primary and metastasis, and RCC from the Institute of Pathology, Technical University of Munich, CCC normal and tumor, Institute of Pathology, Charité, Berlin) to identify relevant cases. The clinical data was provided by the internal clinical data from the respective University Hospitals. In order to allow for high-throughput analysis, selected areas of the tumor tissue of every patient were combined in the TMAs. The study was conducted in accordance with the Declaration of Helsinki. PDAC vs. CCC, CCC tumor vs. normal protocol has been approved by the ethics committee of the Medical University Charité Berlin (application EA1/06/2004). Renal tumors protocol and PDAC primary vs. metastasis has been approved by the review board of ethical committee of the School of Medicine of the Technical University of Munich (Approval 403/17S). CC protocol of was approved by the Institutional Review Board of the University Hospital of Augsburg (Approval 25.09.2018—BKF 2018-18) and performed according to the national rules. The resected tissue was analyzed by hematoxylin and eosin (H&E) staining and areas of interest were marked. Cylindrical tissue cores (0.4–1.0 mm in diameter, 4–8 mm in height) were removed from a specific area of interest within individual ‘donor’ paraffin blocks and relocated in an array-like format into preformed holes, equally spaced 0.5 mm apart, in an empty recipient paraffin block (45 × 20 mm). Tumor tissue used for TMA construction had been initially fixed in 4% buffered formalin and embedded into paraffin. Patient samples were randomly distributed across TMAs. The TMAs were produced according to the standard operation procedure of each institution.

### 4.2. Sample Preparation

From each TMA, a section of 4 μm was adhered to an indium-tin-oxide (ITO) slide (Bruker Daltonics, Bremen, Germany). Sample preparation has previously been described in detail [15,16]. Briefly, sample slides were heated to 80 °C prior to dewaxing with xylene (Carl Roth GmbH, Karlsruhe, Germany), and subsequent rehydration with increasingly concentrated ethanol washes (Carl Roth GmbH, Karlsruhe, Germany). Afterward, the samples were subjected to heat-induced antigen retrieval in MilliQ water at 95 °C for 20 min. A trypsin (Promega, Mannheim, Germany) solution was prepared in 40 mM ammonium bicarbonate (Sigma-Aldrich Chemie GmbH, Munich, Germany) to a final concentration of 0.1 µg/µL. The enzyme solution was sprayed with an automatic sprayer (TM Sprayer, HTX Technologies, Chapel Hill, NC, USA) in 16 cycles with a fixed spraying flow of 150 μL/min. On-tissue digestion was carried out for 2 h at a controlled temperature of 50 °C. Following digestion, four cycles of matrix solution (10 mg/mL of alpha-cyano-4-hydroxycinnamic acid matrix (Sigma-Aldrich Chemie GmbH, Munich, Germany) in 70% acetonitrile aqueous solution with 1% trifluoracetic acid (Carl Roth GmbH, Karlsruhe, Germany)) were deposited with a defined flow of 120 μL/min and a temperature of 75 °C.

### 4.3. Proteomic Characterization by Matrix-Assisted Laser Desorption/Ionization Mass Spectrometry Imaging

MSI was performed using a RapifleX MALDI Tissuetyper time-of-flight (TOF) mass spectrometer (Bruker Daltonics). A peptide calibration standard mix (bradykinin, angiotensin II, angiotensin I, substance P, bombesin, ACTH clip 1–17, ACTH clip 18–39, and somatostatin 28 (Bruker Daltonics)) was used for external calibration. Each spectrum was automatically generated at a spatial resolution of 50 µm using flexControl (Bruker Daltonics) in the mass range of *m*/*z* = 600–3200. 500 laser shots were acquired for each spectrum at 1 kHz, with a laser power of 65–80%. The measurement regions were defined using flexImaging (Bruker Daltonics). Following the MSI measurements, matrix was removed by two washes in 99.99% methanol (Carl Roth GmbH, Karlsruhe, Germany) for 2 min each, followed by two washings in 99.99% ethanol (Carl Roth GmbH) for 10 s.

### 4.4. Tumor Annotation, Data Processing, and Extraction

Following matrix removal, the very same TMA sections measured by MSI, were stained with H&E and digitalized utilizing a slide scanner (Aperio CS2, Leica Biosystems, Nussloch, Germany). H&E scans were uploaded, and tumor regions were thoroughly annotated using SCiLS Cloud (discontinued service from Bruker Daltonics, Bremen, Germany) or QPath (v0.2.2) (Queen’s University, Belfast, United Kingdom) by a pathologist (B.C., B.M., and K.S.) [17]. MSI data was processed using SCiLS Lab Pro (Bruker Daltonics) for mass spectrometry and image visualization. No damage was inferred on the tissue by the MALDI-TOF laser. Annotations were imported into SCiLS Lab Pro software. Spectra baseline was normalized to the total ion count (TIC). Additionally, the spectra were preprocessed for intensity profile normalization, re-sampling, spatial de-noising, and calculation of a second normalization profile [18,19]. Subsequently, automated peak picking with weak de-noising was performed, and spectra of individual spots were exported to .csv- format, and imported to R statistical software (version 3.6.3) (R Foundation for Statistical Computing, Vienna, Austria) via RStudio 1.2.5033 [20,21], for further analysis.

### 4.5. Statistical Analyses—Supervised Classification

The dataset was split into training (70%) and test (30%) sets, with the method control set to 10-fold cross-validation for all models. The classification models were fitted using the “caret” package on R (3.6.3). Linear Discriminant Analysis (LDA) was fitted using the method “lda”. The principle behind LDA is to define a comparable space for data sample of lower dimension in which the data points are “separable”. The data “separability” is based on the mean value and variance. As the solution can be obtained by solving generalized eigenvalues, this model allows for fast processing of large data sets [22]. Random forest classification, simply put, uses a combination of tree classifiers where each classifier is generated using a random vector. A series of decision trees are then evaluated to cast the most popular class and input the vector [23]. Random Forest (RF) was fitted using the method “ranger”, with the number of trees set to 50. Tuning parameter ‘min.node.size’ was held constant at a value of 1. Accuracy was used to select the optimal model using the largest value, but kept the same with and without annotations. Support Vector Machine (SVM) accomplishes the classification by creating, in a higher dimensional space, a plane or hyperplane that optimally separates the data into two categories [24]. This model was fitted using the method “svmLinearWeights”. Accuracy was used to select the optimal model using the largest value. The final values used for the models were cost = 0.25 and loss = L1. The fitted models were used to predict the test data subset. The accuracy value was based on the results of the confusion matrices.

## Figures and Tables

**Table 1:** Classification accuracies of the different supervised classification models using non-annotated renal cell carcinoma samples versus using the same dataset with histological annotations of the tumor region.

RCC (Tumor vs. Normal)	Without Annotations	Annotated	Improvement
LDA	74.92	75.35	0.43
SVM	74.33	75.28	0.95
RF	77.97	78.39	0.42

**Table 2:** Classification accuracies of the different supervised classification models using primary pancreatic ductal adenocarcinoma (PDAC) samples and distant metastasis in direct comparison with the same samples considering the histological annotations of the epithelial regions.

PDAC (Primary vs. Metastasis)	Without Annotations	Annotated	Improvement
LDA	58.67	80.64	21.97
SVM	61.36	84.08	22.72
RF	70.50	91.27	20.77

**Table 3:** Classification accuracies of the different supervised classification models using cholangiocarcinoma (CCC) samples with and without histological annotations.

CCC (Tumor vs. Normal)	Without Annotations	Annotated	Improvement
LDA	82.36	93.41	11.05
SVM	83.41	94.84	11.43
RF	91.52	96.44	4.92

**Table 4:** Classification accuracies of the different supervised classification models using samples from stage I and II colon cancer (CC) with and without histological annotation of the epithelial regions.

CC (Stage I vs. II)	Without Annotations	Annotated	Improvement
LDA	93.54	95.24	1.7
SVM	94.34	95.96	1.62
RF	97.13	96.98	−0.15

**Table 5:** Classification accuracies of the different supervised classification models of pancreatic ductal adenocarcinoma (PDAC) versus cholangiocarcinoma (CCC) samples in direct comparison with the same samples with histological annotations of the tumor regions.

mTMAs (PDAC vs. CCC)	Without Annotations	Annotated	Improvement
LDA	80.86	88.77	7.91
SVM	81.62	90.32	8.70
RF	95.49	96.16	0.67

## Data Availability

The data presented in this study are available on request from the corresponding author. The data are not publicly available due to current project restrictions.

## Supplementary Materials

The following are available online at https://www.mdpi.com/article/10.3390/metabo11110752/s1, Figure S1: Non-annotated regions comparison with annotated regions, Figure S2: Section of the kidney tissue microarray annotations in the H&E scan, Figure S3: Non-annotated regions comparison with annotated regions, Figure S4: Tumor class division of the annotated samples, Figure S5: Section of the pancreatic ductal adenocarcinoma and cholangiocarcinoma mixed tissue microarray annotations in the H&E scan.
