# Supplementary material for: The Impact of Histological Annotations for Accurate Tissue Classification Using Mass Spectrometry Imaging
Source: Metabolites. 2021 Oct 30;11(11):752. doi: 10.3390/metabo11110752 (PMC8624953; doi:10.3390/metabo11110752)
Supplement: Supplementary file 1 [file metabolites-11-00752-s001.zip › metabolites-1426011-supplementary.pdf]

metabolites

Supplementary Materials

Article

The Impact of Histological Annotations for Accurate Tissue Classification using Mass Spectrometry Imaging

Juliana Pereira Lopes Gonçalves [1], Christine Bollwein [1], Anna Melissa Schlitter [1,2], Benedikt Martin [3], Bruno Märkl [3], Kirsten Utpatel [4], Wilko Weichert [1,2,5] and Kristina Schwamborn [1,*]

[1] Institute of Pathology, School of Medicine, Technical University of Munich, Trogerstraße 18, 81675 Mu-nich, Germany; juliana.goncalves@tum.de (J.P.L.G.); christine.bollwein@tum.de (C.B.); melis-sa.schlitter@tum.de (A.M.S.); wilko.weichert@tum.de (W.W.)
[2] German Cancer Consortium (DKTK), Partner Site Munich, Munich, Germany
[3] General Pathology and Molecular Diagnostics, Medical Faculty, University Hospital of Augsburg, 86156 Augsburg, Germany; benediktm@googlemail.com (B.M.); bruno.maerkl@uka-science.de (B.M.);
[4] Institute of Pathology, University of Regensburg, 93053 Regensburg, Germany; Kirsten.Utpatel@klinik.uni-regensburg.de
[5] Comprehensive Cancer Center Munich (CCCM), Marchioninistraße 15, 81377 Munich, Germany
* Correspondence: kschwamborn@tum.de

Figure S1: Non-annotated regions comparison with annotated regions 2
Figure S2: H&E stained detail of the annotated kidney tissue TMA. 3
Figure S3: Non-annotated regions comparison with annotated regions.. 4
Figure S4: Tumor class division of the annotated samples. 5
Figure S5: Section of the pancreatic ductal adenocarcinoma and cholangiocarcinoma mixed tissue microarray annotations in the H&E scan...5

To better illustrate the process of comparison between annotated and non-annotated samples, we have added, one example using the kidney tissue microarray (figure S1). The non-annotated regions, outlined in orange were used for building classification models. The annotated regions, in yellow in figure S1, were independently used to train, validate and test classification models. Both sample sets were subjected to the same algorithms.

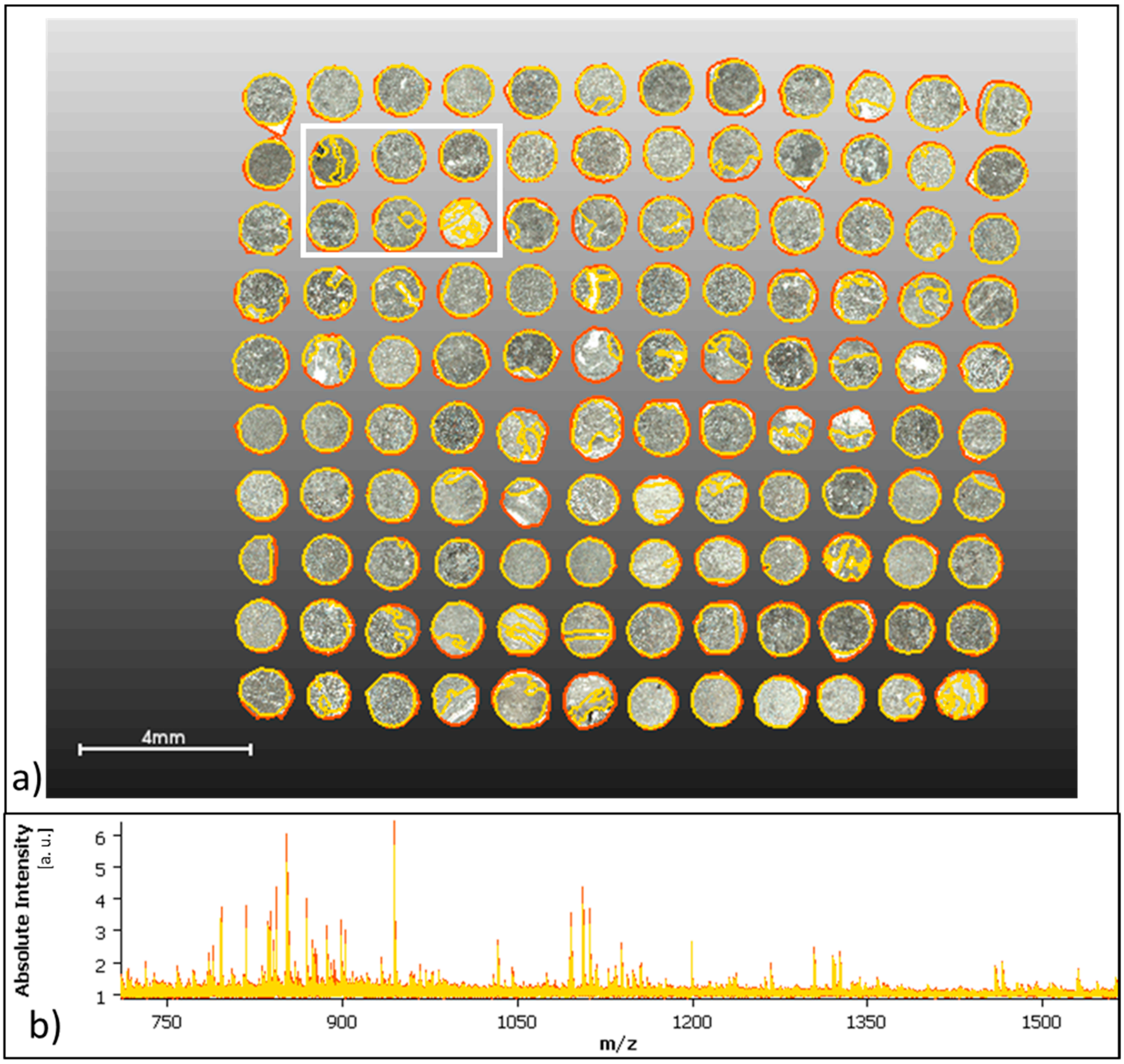

Figure S1. Non-annotated regions compared with annotated regions using SCiLS Lab. (**a**) Kidney tissue microarray, with an overlay of the annotated regions (yellow) over the non-annotated regions (orange). The white rectangle is the section represented in figure S2. (**b**) Segment of the average spectra of the non-annotated regions (orange) comparison with annotated regions (yellow).

To perform the annotations, a pathologist reviews the scan of the hematoxylin and eosin (H&E) stained samples after mass spectrometry imaging measurement. The relevant regions are annotated (figure S2), using a suitable tool, such as QuPath. The annotated regions can then be exported for the data analysis.

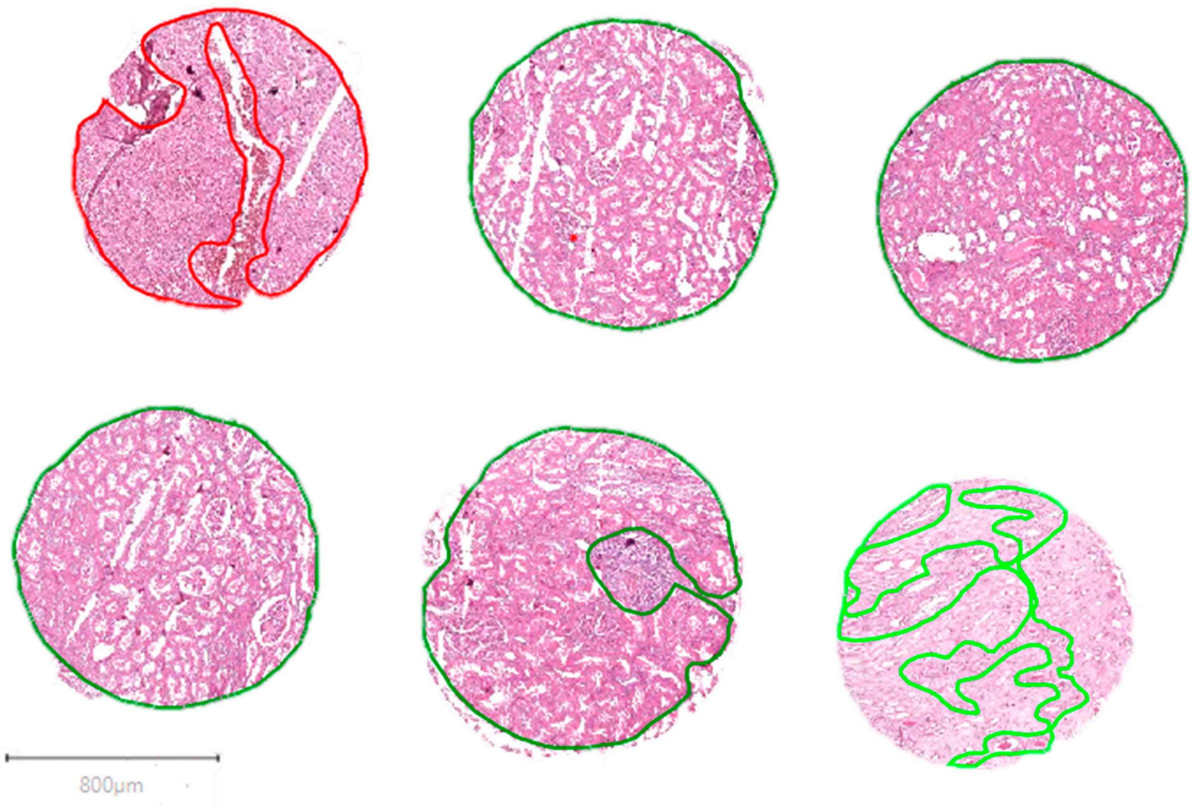

Figure S2. H&E stained detail (as represented by the white box in figure S1) of the kidney tissue TMA showing the histopathological annotations. The tumor regions (red), were annotated after the H&E staining process and scanning of the sample. Normal tissue was marked in green, with dark green representing the renal cortex and lighter green the renal medulla.

Tissue heterogeneity is associated with the tumor location and the bodily function. In figure S3, the detail in the annotations (shown in yellow) represent the heterogeneity of pancreatic ductal adenocarcinoma (PDAC) and the cholangiocarcioma (CCC)

For the classification of the PDAC and CCC mixed TMA, the two different tumor types were defined (figure S4): annotated PDAC is represented in yellow and annotated CCC is represented in orange.

On figure S5, it is well represented the tissue heterogeneity of these tumor types (PDAC and CCC), where some tissue cores have a high density of tumor regions and some have little to no tumor in that particular core.

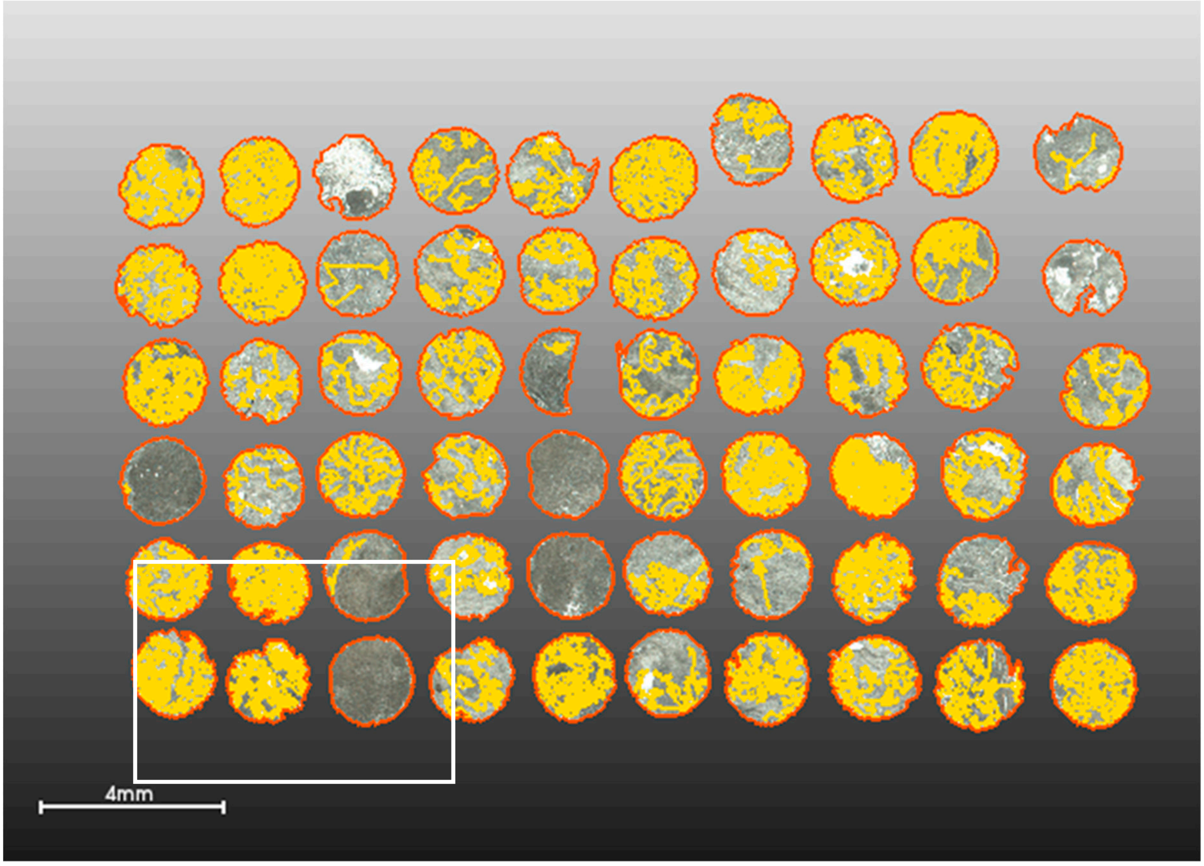

Figure S3. Non-annotated regions in comparison with annotated regions, using SCiLS Lab. Pancreatic ductal adenocarcinoma and cholangiocarcinoma mixed tissue microarray, with an overlay of the annotated regions (yellow) over the non-annotated regions (orange). The white rectangle marks the six cores shown in more detail in figure S5.

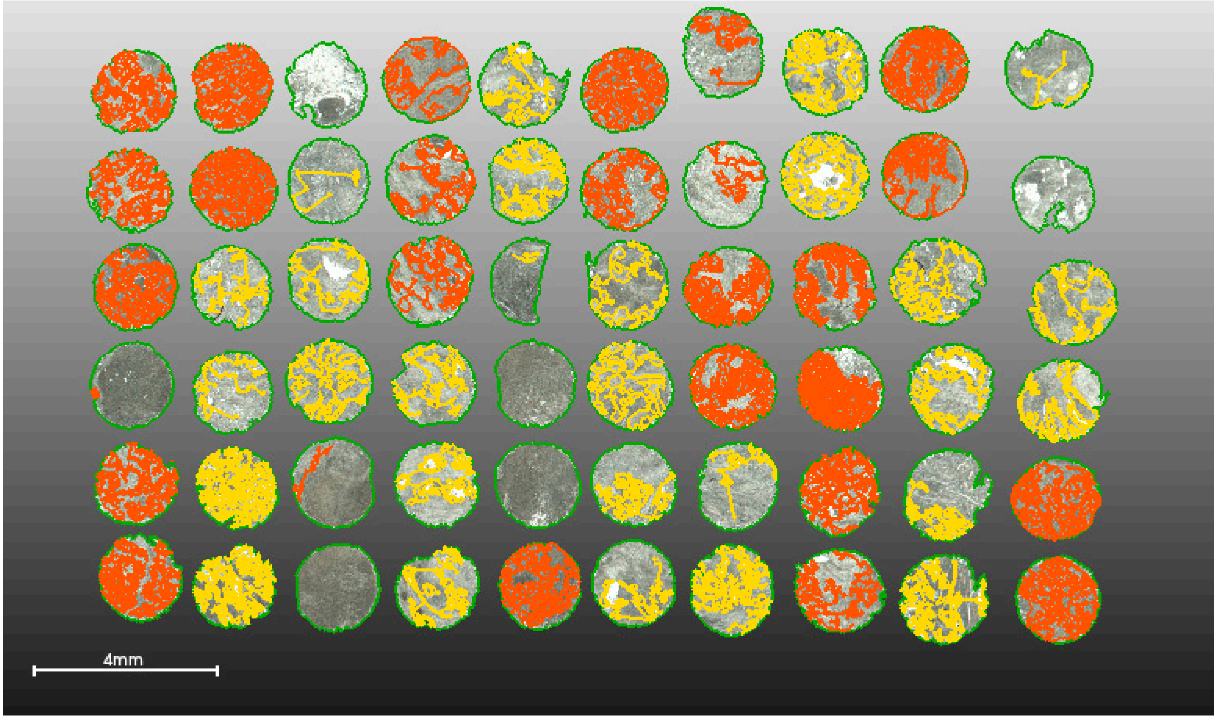

Figure S4. Tumor class division of the annotated samples, using SCiLS Lab. In orange are represented the annotations on the cholangiocarcinoma samples and in yellow the pancreatic ductal adenocarcinoma samples. Measurement regions are outlined in green.

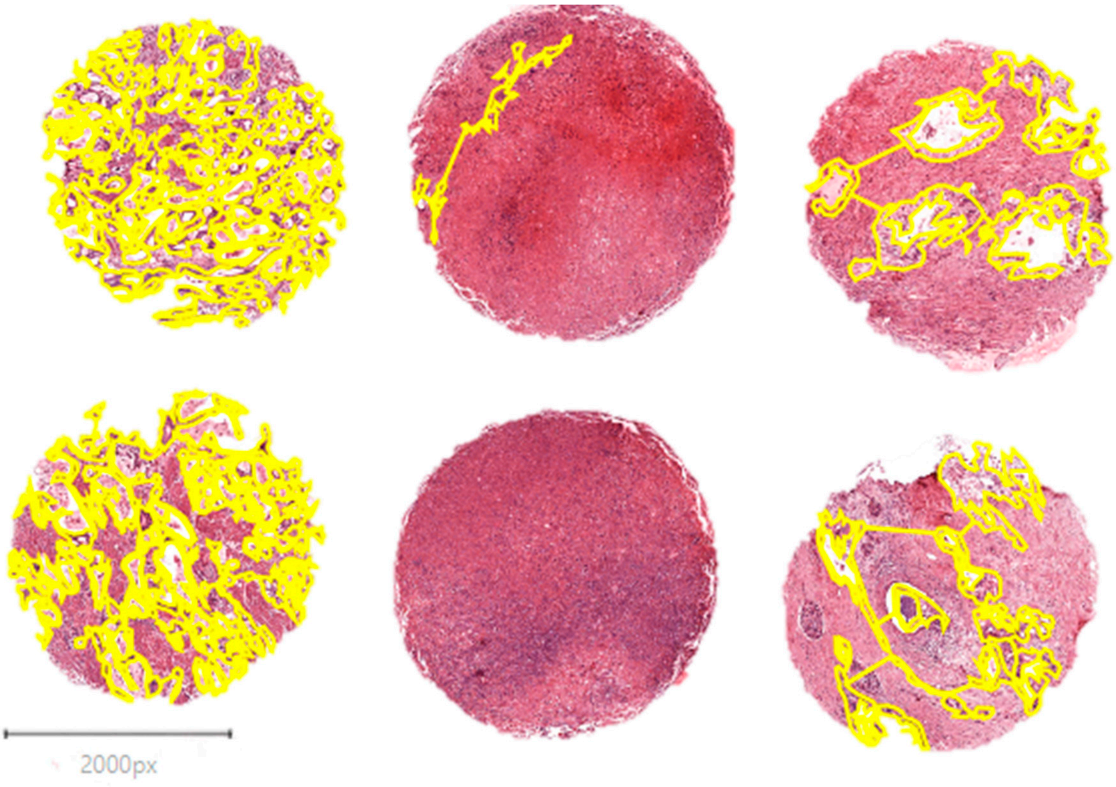

Figure S5. Section of the pancreatic ductal adenocarcinoma and cholangiocarcinoma mixed tissue microarray (represented by the white rectangle in figure S3) with histopathological annotations in the H&E scan. The tumor regions (yellow) were annotated after the H&E staining process and scanning of the sample.
